# Preparing Parents for Discharge from the Neonatal Unit, the Transition, and Care of Their Preterm Children at Home*

**DOI:** 10.17533/udea.iee.v41n1e04

**Published:** 2023-03-14

**Authors:** Sandra Patricia Osorio Galeano, Ángela María Salazar Maya

**Affiliations:** 1 Nurse, Ph.D. Associate Professor. Facultad de Enfermería, Universidad de Antioquia. Colombia. Email: sandra.osorio@udea.edu.co Universidad de Antioquia Facultad de Enfermería Universidad de Antioquia Colombia sandra.osorio@udea.edu.co; 2 Nurse, Ph.D. Full Professor. Facultad de Enfermería, Universidad de Antioquia and Universidad CES. Colombia. Email: angela.salazar@udea.edu.co Universidad de Antioquia Facultad de Enfermería Universidad de Antioquia Colombia angela.salazar@udea.edu.co

**Keywords:** neonatal nursing, infant, premature, patient discharge, intensive care units, neonatal, family, enfermería neonatal, recién nacido prematuro, alta de paciente, unidades de cuidado intensivo neonatal, família, enfermagem neonatal, recém-nascido premature, alta do paciente, unidades de terapia intensiva neonatal, família

## Abstract

Preparing parents to care for their preterm children is one of the principal challenges faced by nursing professionals within the neonatal care contexts. This process seeks for parents to acquire the skills to safely provide the differential care required by children at home given their prematurity condition. The preparation for discharge is complex and multidimensional, involving aspects that have to do with the knowledge, skills for caring, security, and trust to transit and take care of the children at home. This process is conducted in the neonatal unit gradually, in function of the clinical evolution of the children and the adaptation of the parents to the situation, considering their individual, family, social, and cultural characteristics. This article describes the principal aspects related with the preparation for discharge and the transition to the home, contents of the education that must be provided to the parents or principal caregivers, and the recommendations for the professional practice regarding these types of educational processes, aimed at making visible and facilitating the nursing role and follow up of results in the health and wellbeing of the preterm children, their parents, and families.

## Resumen

La preparación de los padres para el cuidado de sus hijos prematuros es uno de los principales retos que enfrentan los profesionales de enfermería dentro de los contextos de cuidado neonatal. Este proceso busca que los padres adquieran la competencia para brindar de forma segura los cuidados diferenciales que requieren los niños en el hogar dada su condición de prematurez. La preparación para el alta es compleja y multidimensional, involucra aspectos que tienen que ver con los conocimientos, las habilidades para el cuidado, la seguridad y la confianza para transitar y cuidar a los hijos en el hogar. Este proceso se desarrolla en la unidad neonatal de forma gradual, en función de la evolución clínica de los niños y la adaptación de los padres a la situación, considerando sus características individuales, familiares, sociales y culturales. En el presente artículo se describen los principales aspectos relacionados con la preparación para el alta y la transición al hogar, los contenidos de la educación que debe brindarse a los padres o cuidadores principales y las recomendaciones para la práctica profesional en torno a este tipo de procesos educativos, tendientes a visibilizar y facilitar el rol de enfermería y el seguimiento de los resultados en la salud y el bienestar de los niños prematuros, sus padres y familias.

## Resumo

A preparação dos pais para o cuidado de seus prematuros é um dos principais desafios enfrentados pelos profissionais de enfermagem em contextos de cuidados neonatais. Esse processo busca que os pais adquiram competência para prestar, com segurança, os cuidados diferenciados que a criança requer em casa, dada a sua condição de prematuridade. A preparação para a alta é complexa e multidimensional, envolve aspectos que têm a ver com conhecimento, habilidades de cuidado, segurança e confiança para transitar e cuidar da criança no domicílio. Esse processo é desenvolvido gradativamente na unidade neonatal, dependendo da evolução clínica da criança e da adaptação dos pais à situação, considerando suas características individuais, familiares, sociais e culturais. Este artigo descreve os principais aspetos relacionados com a preparação para a alta e transição para casa, os conteúdos da educação que deve ser prestada aos pais ou cuidadores principais e recomendações para a prática profissional em torno deste tipo de processo. educativa, tendente a tornar visível e facilitar o papel da enfermagem e o acompanhamento dos resultados na saúde e bem-estar do prematuro, seus pais e familiares.

## Introduction

Preterm delivery, defined as that which occurs before week 37 of gestation, conditions important risks in the health and lives of preterm children, mainly in those with low weight and gestational age at birth, who due to the immaturity of their organs and systems require specialized care within the neonatal unit,^1^ However, these risks do not end with the hospitalization, given that after discharge preterm children continue having high vulnerability and the care they receive at home results determinant in their wellbeing in the short, mid, and long term.^2^ It is important to highlight that caring for preterm children at home supposes some particularities that make it different from caring for term newborns. Parents must learn to care for their children, with the help of the health staff, principally from nursing who plays a leading role in the educational processes related with the preparation for discharge.^3^ Before going home with their children, parents must acquire knowledge, skills and trust for care,^4^ which becomes an important criterion for discharge from the neonatal unit.^4^^-^^6^ This is a complex process, given that the premature birth, hospitalization in the neonatal unit, transition to the home, and care after discharge are critical events for the parents,^7^ who tend to experience high levels of stress, anxiety, guilt, fear, and uncertainty.^8^ These feelings can limit their participation in the process and affect their capacity to care for the preterm children. 

Thus, the preparation for discharge and home care of preterm children are themes of great importance and interest for nursing professionals. Although the core is constituted by knowledge and skills for caring,^5^^,^^8^ the process involves aspects that have to do with preparing for the transition to the new care setting, trust, security, empowerment, and family and social support. Considering these aspects within the process is essential to achieve positive results in the health and wellbeing of children, their parents, and families; among which are highlighted lower numbers of readmissions and consultations, shorter hospital stays, and lower levels of stress and anxiety for the parents.^9^^-^^11^ These aspects reiterate the importance of educational processes aimed at the preparation for discharge in neonatal units and place them as a key component of family-centered care, which promotes involving the fathers in caring for their children to favor the bond and establishment of the paternal role, empowerment for care, communication based on reciprocity and respect, unrestricted presence of the fathers, and - overall - more humanized care.^12^^,^^13^ This work reviews general aspects of the preparation process for discharge and the transition to the home of parents with their preterm children, the contents of the education that must be provided within the process and the key strategies for the professional nursing practice within said process. 

## Characteristics of the preparation for discharge from the neonatal unit

Preparing for discharge is a process characteristic of hospitalization contexts that seeks for patients and their families to feel prepared to leave the hospital and face the care needs at home.^14^ It is closely related with educational processes that promote direct participation in caring prior to the hospital discharge with the accompaniment, support and supervision of the health staff. It is an interdisciplinary process where leadership and participation by nursing have been described as predictive elements in its success.^3^ Within the specific context of the neonatal unit, preparing for discharge supposes obtaining skills and technical knowledge, emotional comfort and trust in caring for preterm children upon discharge.^15^ This process also aims to facilitate the transition to the home by the parents^4^^,^^15^ who must assume the care of their children independently away from the accompaniment of the nursing staff characteristic of the hospital context, which is in itself a critical process.^8^

It should be noted that this is a continuous process that starts at the moment of the premature newborn’s admission to the neonatal unit^4^^,^^15^ and includes formal and informal education.^14^ Parents learn from the routine, the context, and from the nursing practice, even when there is no educational intention, which reaffirms the importance of a professional nursing practice aware and reflective of the process. At the beginning of the hospitalization, the preparation for care must focus on recognizing the dynamics of the neonatal unit, the characteristics of prematurity, and other general aspects of care; given that during the first days, the health and survival situation may be uncertain. Moreover, the parents tend to be confused,^7^^,^^16^ given that the preterm delivery is removed from the social ideal of a child’s birth and confronts them with an unexpected and unknown reality;^16^ home care is a scenario still distant from their reality.

Thereafter, as the premature child reaches a given physiological stability, direct contact with the parents starts, which favors attachment, strengthening of the parental role and educational process related with knowledge and skills for caring for the premature child. It is important to highlight the importance of direct involvement with their care within the unit under the guidance and supervision of the nursing staff; it looks to have the parents achieve a gradual, active, conscious, and informed participation in the educational process.^16^ Assessment of the individual, family, and social characteristics is a central aspect to guide the educational actions, from an individualized perspective that responds to the specific needs of the parents and families.^8^^,^^17^ This perspective reaffirms that preparation for discharge is a process that goes beyond the informative, it is interactive and relational. 

Once the parents became familiar with the reality of caring for their premature child within the neonatal unit, direct care is favored in an increasingly independent manner, promoting the development of security and trust for caring.^16^ Emphasis is placed on the process of each child, on their needs and specific conditions. Guidance is also provided about the transition process, offering realistic information about the challenges they will face at home, mainly during the first week after discharge.^4^ Likewise, the dynamics of the monitoring programs are revealed, as well as the importance of family and social support. In this sense, it is very important to extend, whenever possible, the information and preparation to other significant people in the family, so that they can provide to the parents support close to their reality and to the needs of the preterm children; this involvement can be conducted directly or indirectly through virtual means,^4^ in case of having restrictions for admission to the neonatal unit. In all cases, it is expected for the families to be sensitive and receptive to the needs of the parents and of their preterm children at home. 

The principal *characteristics of the preparation process for discharge from the neonatal unit and the transition to the home* are listed:

- Starts since admission to the neonatal unit.
- It is a relational, continuous, and dynamic process.
- Involves formal and informal education.
- Recognizes the individual, family, and social conditions of the parents or caregivers.
- It is framed within the specific conditions and needs of each premature child.
- Advances according with the child’s clinical evolution and the parents’ adaptation to the situation.
- Includes contents related with physical care, emotional preparation to develop trust and security.
- Considers and favors the successful transition to the home.
- Must be extended directly or indirectly to the family.

## The transition of parents and their preterm children to the home

The transition to the home by the parents with their preterm children has been described as a critical process, which must be considered among the preparation processes for discharge.^4^^,^^8^ Separation from the infant, limitations to fulfill the parental roles during hospitalization, stress, anxiety, and uncertainty can diminish trust for caring and make the transition to the home a specially complex event.^8^^,^^18^^-^^22^ It is characterized for being a cyclical process, no-lineal, not limited over time, of accepting responsibilities by the parents when going from the security of the hospital environment to the independent care at home for their still vulnerable children, far from the monitoring and continuous accompaniment from the nursing staff.^8^^,^^21^^,^^22^

What makes the transition from hospital to home specially complex for the parents of preterm children is that they experience at least two transitions; one is the transition to paternity and two the transition from hospital to home.^7^^,^^19^^-^^23^ This situation confronts them with big challenges and exposes them anew to high levels of stress, fear, and uncertainty.^8^^,^^21^ Add to all this the burden of care demanded by their child, particularly those going home with continuous kangaroo method until reaching the weight goals proposed to suspend such. 

For the transition to the home, the parents must make adjustments related with the needs of their children in terms of hygiene, temperature, supplies, and devices for care, among others,^4^ but also in the space and physical conditions. Home care decisively involves social relationships and support networks that are part of daily life and which facilitate the task of caring. Social support is a determining factor in the transition to the home.^23^^-^^26^ At home, relatives, neighbors, and other significant people become a support for caring and their influence regarding care may be both positive or negative, given that in some cases, care for a premature child is unknown and questioned by some family members,^26^ thus, the importance of involving them in the preparation process for discharge and guiding their support so that it facilitates the tasks of the parents and helps them during the complex transition process they must undertake after discharge from the neonatal unit. 

## Contents addressed during the preparation process for discharge and the transition to the home

Within the complexity and multidimensionality of the process, knowledge and skills for caring are central aspects,^8^^,^^21^ around which revolve phenomena as sensitive and important as the enhancement of the parental role and bond,^27^ attachment, empowerment, and trust for care.^14^^,^^16^ Guidelines currently exist on the theme, ^(^^4^^,^^6^^,^^28^ which permit specifying the contents that must be addressed within the processes of preparing the parents for discharge from the neonatal unit and that, being based on the particularities of preterm children, permit giving continuity to the care and guarantee adequate growth and development after discharge. The goals of the process must be individualized, but the common contents may be grouped as: general information of prematurity, care related with skin-to-skin contact through the kangaroo method, maternal breastfeeding, and feeding of the premature child at home, hygiene, general and specific care, warning signs, basic reanimation, and preparation for the transition to the home. 

*General information of the prematurity.* Parents must be notified about the prematurity, its causes, risks, and implications. This information must be offered early and aimed at recognizing the need for differential care with respect to term children, to favor active and conscious participation of the parents in the process^4^. Acknowledging that their child requires at home, for some time, specific care to guarantee adequate growth and development is a strong motivation for participation and learning.^8^ Among the aspects that must be introduced to the parents, there are the classification of prematurity according to gestational weight and age at birth, appearance and behavior, thermoregulation of the premature child, nutritional requirements, need for support and stimulation for suction and feeding, physiological characteristics derived from the immaturity of organs and systems, susceptibility to infections, among others. This general information is given regardless of the time of discharge, which in many cases cannot be anticipated and overall seeks greater comprehension of the situation by the parents and anticipated comprehension of the need for their active participation in caring for the premature child within the neonatal unit, as principal players in the process in which they will gradually have greater responsibilities of care.^4^^,^^6^^,^^28^

***Skin-to-skin contact through the kangaroo method*.** One of the most important aspects of caring for preterm children weighing less than 2,500 g at birth has to do with skin-to-skin contact through the kangaroo method. This specific care tends to be conducted with the parents in the neonatal unit when the child’s conditions so permit, alternating with time in the incubator; besides, it is care that the parents or caregivers must provide continuously or intermittently after discharge according to the weight on discharge. The method was first described in Colombia in 1970 and since then has been performed and has provided evidence throughout the world about its positive effects for children and their parents.^29^ It consists in maintaining the premature child on the chest of the parent or caregiver, guaranteeing skin-to-skin contact between the parent’s chest and the child’s abdomen.^29^^,^^30^ The child must remain in vertical position, with the head turned to one side and slightly extended to favor breathing; the legs must remain apart. To maintain the position, an elastic sash is used that surrounds the caregiver's torso and the child must remain with a cap and stockings to avoid loss of temperature through these surfaces. The length of permanence depends on the weight at discharge; if in continuous manner, the premature child should only be removed for diaper change and to go from one caregiver to another. Feeding must take place within the method. The method permits early discharge; the heat provided by the caregiver avoids spending the child's limited reserves to regulate their temperature, so they will have better weight and height gains.^29^^-^^31^ Contact with the breath and the heartbeat of the parents or caregivers stimulates their breathing and favors the affective bond. Performing the method in the neonatal unit with guidance and support from the nursing staff contributes to the parents feeling more secure to perform it at home.^4^ Parents must be informed about the steps of the kangaroo method from continuous to intermittent; its ending depends on the weight gain and will be informed during follow up after discharge. 

***Maternal breastfeeding and feeding of the premature child at home*.** It includes all the aspects related with the maternal breastfeeding, which is promoted as the best feeding alternative for newborns and preterm children.^32^^,^^33^ This component must reinforce its advantages, feeding techniques, extraction, and conservation.^33^ However, it is important to highlight the specifics of feeding preterm children. Many of them, during hospitalization, are fed initially fed through a catheter, given the characteristic immaturity of their suction and swallowing mechanisms, added to other possible clinical conditions. During hospitalization, the mothers start the process of feeding their children gradually and can face situations, like low production of breastmilk and weak suction by their children, which complicates the breastfeeding experience.^33^ In this sense, it is important to teach stimulation techniques for suction and administration of breastmilk extracted with a syringe or cup. The frequency of feeding must also be indicated, according to the weight upon hospital discharge and the importance of being persistent in this task that represents big challenges for parents of preterm children. 

***Hygiene and general care*.** These types of contents focus on guiding parents and caregivers to recognize the importance of frequent diaper changes to avoid prolonged humidity, promote skin care, hygiene through a sponge bath, and limiting the possibilities of temperature loss.^4^^,^^8^ The time to perform the immersion bath will be informed during the follow-up and will be decided by the weight gain. These contents also have to do with cleaning of the oral cavity and nasal washing. This last content stems from knowing that children are nose breathers and any obstruction could cause some respiratory alteration, so emphasis in this sense must be reiterated and parents must acquire the skill and trust to perform this type of care. 

***Specific care*.** Some preterm children may remain for some time after discharge with home oxygen, which implies educating the parents on managing the oxygen at home. These situations tend to be temporary and their duration will depend on the situations identified during follow up. In this same sense, if the child is discharged with medications, the parents should be taught about their indications, administration, and effects.^4^ In addition, particular situations, like ostomies, wounds, catheters, or other special situations require specific educational processes. 

***Warning signs*.** Parents must be able to recognize at home situations that indicate existence of any alteration in the wellbeing of their children, anticipate complications, and make timely inquiries.^4^ For such, they must be informed about the warning signs that may indicate the need for intervention and consultation. It is useful to set out warning signs for systems, clearly, in a language that is comprehensible to them.^4^ For example, at respiratory level, it includes labored, rapid breathing, flaring or twitching of the nostrils, persistent grunting when breathing, bluish color in the mouth and fingers, and pauses in breathing. On the skin, paleness, a purple or yellow tonality. At the behavioral level, the warning signs are excessive crying for no apparent reason, staring without response, abnormal movements, rigid or slack position, and weak or no suction. At the gastrointestinal level, distended abdomen, frequent vomiting, bloody stools, among others. Parents should be invited to locate the nearest consultation place and the means of travel to reach said place quickly in case it is required. 

***Basic reanimation*.** Within the preparation for home care, the parents should be taught to identify respiratory failure and initiate basic reanimation maneuvers, as a measure to save the lives of children that could suffer this type of event.^4^^,^^8^^,^^29^ Guidance must be adjusted to guidelines in effect for lay resuscitators.^34^

***Transition and arrival to the home*:** the hospital discharge is more a process than a specific moment, the interdisciplinary team participates in the process and assesses the parents’ competence for home care. The nursing staff together with the parents must plan the arrival to the home, guide about the preparation of the physical space, devices and elements for care, and people who will support them during home care. Similarly, it must confirm the viability of complying with follow up appointments, travel to controls and other logistic aspects for care.^4^^,^^21^^,^^22^ In addition, parents must be realistically informed about the challenges they will face at home derived from the high burden of caring for their premature child,^4^ principally the first weeks after discharge. They will be urged to express their emotions, to talk about them with the health staff and believe in their capacities to cope with the situation, which, although complex and demanding, is temporary. These aspects are very important within the process and seek to favor the parents’ empowerment and trust. It should be highlighted that the contents must be delivered within the framework of an interpersonal relationship of trust that considers parents as active and central subjects within the process. Their involvement with the direct care of their child during hospitalization is the strategy of greatest importance because it favors attachment and helps them to gain security and trust regarding their capacity of caring.^27^^,^^28^

## Recommendations for the professional practice in nursing within the preparation processes for discharge from the neonatal unit

As described, the nursing role within the processes of preparing for discharge from the neonatal unit and the transition to the home is determinant in its success, in the results on the health of the children, and in the satisfaction of the parents and families.^3^ This has been a topic of interest from research and the experience of neonatal nursing, which is why it is possible to establish recommendations that can be applied to the professional practice to favor the quality of these processes. 

In this respect, one of the principal recommendations has to do with the need to structure the formal component of the process^3^^,^^35^, define the educational actions, minimum times, number of formal sessions and contents according to the different moments, as well as the process to assess the competence for care before discharge. Formalization helps to make visible what nursing is to do within the processes of preparing for discharge, clarifies its role within said processes, and facilitates follow up and achievement of results.^36^ Likewise, within the formalization of the program, it is important to involve disciplinary theoretical elements^36^ that facilitate the delivery of the contents in function of the needs for coping, adaptation, empowerment, intercultural care, among others,^37^^,^^38^ within the overall framework of the family-centered care model.^39^

It should be noted that the perspective of intercultural care is an important theme in preparation processes for discharge; phenomena, like cultural diversity in each country and migration in different contexts globally are examples of the importance of considering this approach.^40^ Caring for and raising children, in general, is social knowledge transmitted from generation to generation, but care for preterm children requires differential practices that are not known by communities. The preparation for discharge from the neonatal unit must start from recognizing the social and cultural reality of the parents and families, to generate agreements and tend toward consistent and informed care about the needs and particularities of the preterm children. It is imperative to have a culturally respectful, individualized, and family-informed approach that considers diversity inclusion and equity of care, care, and education.^4^ Moreover, it is also recommendable sensitize and coordinate the entire nursing staff with the process and goals for the parents and families, bearing in mind their specific conditions. Education is a relational process of reciprocal interaction and, hence, in daily care, all interactions with the parents are constituted as educational opportunities; many of the daily actions by nursing, like listening, providing words of encouragement and motivation, are part of the strategies for the emotional preparation of the parents, from the informal component of the process. Parents observe and learn from the nursing staff, even when there is no intentionality of the educational act, therein the responsibility of the entire staff in the preparation for discharge. 

Another important recommendation is related with the development of educational material, like booklets, videos, sheets, applications, and virtual contents.^41^ These aids facilitate the educational process and become support material for the nursing staff and consultation source for the parents and the family. It is necessary for the material to be concrete, simple, close to the language of the parents, friendly in its presentation, illustrative, with social and cultural pertinence for which it is recommendable for the program to develop its own material or suggest to parents to consult pages or sources of information, previously verified in compliance with these conditions. In this same sense, it is recommended to use virtual media to complement educational processes. This has been a theme of special relevance, particularly during the COVID-19 pandemic,^42^ where these tools were the only means possible to maintain contact with the parents. In this sense, the convenience of virtual media is reaffirmed in educational processes aimed at parents of preterm children; however, it is clear that the face-to-face interaction of the parents with the nursing staff, the bond, and direct care of the child in the unit are irreplaceable. 

The presence and permanence of parents in the neonatal unit is one of the principles of family-centered care and, in turn, a determining factor for parents to enhance their knowledge and skills, participate in decision making, exercise their paternity, develop trust and security.^12^^,^^13^ Within the unit, the parents must receive direct information, resolve concerns, and get the help and emotional support they need. The demonstration of care, their execution with accompaniment and guidance from the nursing staff makes possible increasingly independent care in the unit as the discharge nears, which facilitates the transition. Likewise, it is very important to extend the education to significant family members to promote social support closer to the realities of prematurity and to the needs of the parents. This facilitates the arrival to the home and eases the high burden of care and concerns of the parents. It has been described that some of them can avoid having other people help them because they fear that they can provide inadequate care, but when the family participates in the process and receives direct information from the health staff, the parents feel more confident regarding the family support in caring for their children.^43^

Moreover, participation and leadership by nursing in the monitoring programs are among the most important recommendations for the professional practice,^3^ given that such favor the continuity of care and of the very educational process, aimed at this moment of care at strengthening the capacities and skills for caring and to monitoring the conditions of health, growth, and development of preterm children. 

Finally, it is highly relevant to conduct research processes tending to evaluate the effect of formal educational processes on the satisfaction of the nursing staff and of the parents; as well as on the health and wellbeing of the preterm children. It is also necessary to explore and know other phenomenon of interest in the neonatal care contexts from the voices of the different players to broaden understanding the reality of the parents and families of preterm children, recognizing the cultural and social particularities to contribute to the disciplinary knowledge, its application in the professional practice and to facilitate decision making in neonatal care processes. 

The following lists the *recommendations for the professional nursing practice* in neonatal units to prepare parents to care for their children at home after the hospital discharge:

- Formalize the preparation process for discharge in their structure, delivery of information, contents, evaluation, and follow up.
- Guide the process and delivery of contents within the general framework of family-centered care.
- Extend the educational process for discharge, transition and home care to other significant family members.
- Involve disciplinary theory to guide the approach of phenomena of interest.
- Favor the intercultural approach, inclusion, and respect for diversity.
- Coordinate and involve the entire nursing staff with respect to the process.
- Develop own educational material, which responds to the cultural and social context.
- Use virtual media to support and complement face-to-face educational processes within the neonatal unit.
- Conduct research processes to assess the effect of educational processes on the health of the children and satisfaction by the parents and health staff and to broaden comprehension of the phenomena implied in the process, to generate evidence that facilitates decision making in the neonatal care contexts.

## Conclusion

Preparing parents of preterm children for discharge from the neonatal unit, the transition and home care is a complex and multidimensional process in which nursing plays a central role. The principal characteristics of this process have to do with its continuity and early start, even since admission to the unit, its recognition as a relational, gradual, and dynamic process, which seeks for parents to acquire the knowledge, skills, emotional comfort, security, and trust to transit to the home and care for their preterm children in a safe and competent manner. The contents of this educational process are diverse and are based on recognizing the vulnerability characteristic of the immaturity of the organs and systems of children born prematurely. Overall, the contents may be grouped into the general information of prematurity, skin-to-skin contact through the kangaroo method, maternal breastfeeding and feeding the premature child at home, hygiene and general care, specific care, warning signs, basic reanimation, and the transition and arrival to the home. 

All these contents must be delivered within the framework of an interpersonal, interactive relation in which parents play an active role in the direct care of their child, within a context of family-centered care. Among the current recommendations for the nursing practice in preparation processes for discharge, the need is highlighted to formalize processes, generate a commitment by the entire nursing staff, consider the individual, social, and cultural perspectives of each family, and articulate the disciplinary theory to address the phenomena of interest experienced by parents and families of preterm children. It is also important to extend the process to significant people to the parents to favor the transition and social support, as well as develop and use educational material and employ virtual media to support and complement the process. Finally, the need is restated to conduct research processes to broaden evidence regarding the theme and guide decision making in neonatal care contexts.
